# Chemical Composition of *Piper nigrum* L. Cultivar Guajarina Essential Oils and Their Biological Activity

**DOI:** 10.3390/molecules29050947

**Published:** 2024-02-21

**Authors:** Bruna de Souza Feitosa, Oberdan Oliveira Ferreira, Celeste de Jesus Pereira Franco, Himani Karakoti, Ravendra Kumar, Marcia Moraes Cascaes, Rahul D. Jawarkar, Suraj N. Mali, Jorddy Neves Cruz, Ilmarina Campos de Menezes, Mozaniel Santana de Oliveira, Eloisa Helena de Aguiar Andrade

**Affiliations:** 1 School of Chemistry, Federal University of Pará, Rua Augusto Corrêa S/N, Guamá, Belém 66075-900, PA, Brazil; brunaufpa08@gmail.com (B.d.S.F.); celeste.franco12@hotmail.com (C.d.J.P.F.); eloisa@museu-goeldi.br (E.H.d.A.A.); 2 Graduate Program in Biodiversity and Biotechnology—Rede Bionorte, Federal University of Pará, Rua Augusto Corrêa S/N, Guamá, Belém 66075-900, PA, Brazil; oberdan@museu-goeldi.br (O.O.F.); jorddynevescruz@gmail.com (J.N.C.); 3 Department of Chemistry, College of Basic Sciences and Humanities, Govind Ballabh Pant University of Agriculture and Technology, Udham Singh Nagar, Uttarakhand 263145, India; himanikarakoti@gmail.com (H.K.); ravichemistry.kumar@gmail.com (R.K.); 4 Graduate Program in Chemistry, Federal University of Pará, Rua Augusto Corrêa S/N, Guamá, Belém 66075-900, PA, Brazil; cascaesmm@gmail.com; 5 Department of Medicinal Chemistry and Drug Discovery, Dr. Rajendra Gode Institute of Pharmacy, University Mardi Road, Amravati 444603, India; rahuljawarkar@gmail.com; 6 School of Pharmacy, D.Y. Patil University (Deemed to be University), Sector 7, Nerul, Navi Mumbai 400706, India; mali.suraj1695@gmail.com; 7 Embrapa Amazônia Oriental, Tv. Dr. Enéas Pinheiro, s/n-Marco, Belém 66095-903, PA, Brazil; ilmarina.menezes@embrapa.br; 8 Adolpho Ducke Laboratory—Coordination of Botany, Museu Paraense Emílio Goeldi, Av. Perimetral, 1901, Terra Firme, Belém 66077-830, PA, Brazil; 9 Programa de Pós-Graduação em Ciências Biológicas—Botânica Tropical, Universidade Federal Rural da Amazônia, Museu Paraense Emílio Goeldi, Av. Perimetral, 1901, Terra Firme, Belém 66077-830, PA, Brazil

**Keywords:** natural products, *Piperaceae*, Amazon, essential oil, antioxidant activity, toxicity *Artemia salina*

## Abstract

The essential oils and aroma derived from the leaves (L), stems (St), and spikes (s) of *Piper nigrum* L. cv. Guajarina were extracted; the essential oils were extracted using hydrodistillation (HD), and steam distillation (SD), and the aroma was obtained by simultaneous distillation and extraction (SDE). Chemical constituents were identified and quantified using GC/MS and GC-FID. Preliminary biological activity was assessed by determining the toxicity against *Artemia salina* Leach larvae, calculating mortality rates, and determining lethal concentration values (LC_50_). The predominant compounds in essential oil samples included α-pinene (0–5.6%), β-pinene (0–22.7%), limonene (0–19.3%), 35 linalool (0–5.3%), δ-elemene (0–10.1%), β-caryophyllene (0.5–21.9%), γ-elemene (7.5–33.9%), and curzerene (6.9–31.7%). Multivariate analysis, employing principal component analysis (PCA) and hierarchical cluster analysis (HCA), revealed three groups among the identified classes and two groups among individual compounds. The highest antioxidant activity was found for essential oils derived from the leaves (167.9 41 mg TE mL^−1^). Larvicidal potential against *A. salina* was observed in essential oils obtained from the leaves (LC50 6.40 μg mL^−1^) and spikes (LC_50_ 6.44 μg mL^−1^). The in silico studies demonstrated that the main compounds can interact with acetylcholinesterase, thus showing the potential molecular interaction responsible for the toxicity of the essential oil in *A. salina*.

## 1. Introduction

The Piperaceae has a wide distribution in tropical and subtropical regions of the Northern and Southern Hemispheres. This family comprises approximately 3600 species that are distributed in herbaceous plants, shrubs, herbs, subshrubs, epiphytes, and rupicolous or terrestrial plants [1,2]. Furthermore, within this botanical family, there are five genera: *Macropiper*, *Zippelia*, *Piper*, *Peperomia* and *Manekia* [2]. The genus *Piper* L. has approximately 2000 species, which can be easily identified in collection excursions because they have some peculiar characteristics, such as knotted shoots, tips of inflorescences, and a spicy or aromatic smell [3]. Some species of this genus are used in traditional medicine as analgesics for the treatment of pain, such as in toothache and wound treatment [4].

*Piper nigrum* L., “pimenta-do-reino”, is described as a species of perennial vine that grows in the presence of shade from other trees or support poles. The leaves of this species are botanically simple, long (8–20 cm), wide (4–12 cm), and alternating, with a furrowed petiole 2 to 5 cm long [5]. In addition, this species produces essential oils (EOs) in both leaves and fruits, which have antioxidant and biological properties such as antimicrobial effects, cytotoxicity, insecticidal effects, and anti-inflammatory toxicity [5,6,7].

The antioxidant properties of the EOs of *P. nigrum* L. have strong potential to reduce the harmful effect of free radicals, which are produced by an imbalance of reactive oxygen species (ROS) in the body. Free radicals cause serious problems to almost all components of the cell, including proteins, lipids, and DNA [6]. The toxicity properties of *P. nigrum* L. indicate great potential for the production of natural biopesticides due to its biodegradability, and studies of this species have proven the effectiveness of its EO [8]. In addition, it is important to seek to understand the mechanism of action of bioactive compounds, in relation to preliminary toxicity. A method widely used by several researchers is with *Artemia salina*. It is also reported that the probable molecular target is an acetylcholinesterase, (AChE) is an enzyme that breaks down the neurotransmitter acetylcholine (ACh) in the nervous system. It is found in various organisms, including the brine shrimp [9]. The *A. salina* is a small, aquatic crustacean commonly used as a model organism in scientific research. The AChE in *A. salina* is an important target for neurotoxicity testing because it is highly sensitive to many environmental pollutants, such as heavy metals and pesticides. Researchers can measure the activity of AChE in *A. salina* as an indicator of the potentially toxic effects of various substances. For example, if a substance inhibits the activity of AChE in *A. salina*, it may be a neurotoxin and could be harmful to other organisms, including humans [10,11,12,13]. This study is of paramount importance as it addresses gaps related to *P. nigrum* L., specifically the Guajarina cultivar. These gaps encompass the chemical composition of the essential oil, its antioxidant potential, and toxicity. Popularly known as black pepper, it serves not only as a seasoning in food but also as a culinary spice, imparting properties that preserve meats and lend them a pleasant flavor characterized by its pungency. This attribute stimulates increased salivary flow and gastric juices, enhancing the palatability of foods. Furthermore, these cultivars are employed as a means of enhancing the plant, making it more resistant to pests such as phytopathogens. Therefore, the research aims to address crucial gaps related to this specific cultivar, contributing to a comprehensive understanding that spans from chemical aspects to practical implications in culinary applications and the plant’s resilience to adversities. Thus, the objective of this study was to study the chemical composition, antioxidant potential, and preliminary toxicity of Eos. In addition, this work presents an in silico study of the probable mechanisms of action of the major compounds on AChE, aroma and essential oil from leaves, stems, and spikes of the species *P. nigrum* cv Guajarina.

## 2. Results and Discussion

### 2.1. Yields of Essential Oils

The yields of *P. nigrum* cv Guajarina EOs obtained from the leaves (L), stem (St) and spike (s) by hydrodistillation (HD) and steam distillation (SD) in November (nov) and March (mar) ranged from 0.29 to 1.96%. Our results were very close to those observed in the study by Li et al. [14] on a specimen of *P. nigrum* collected in China. Regarding the samples extracted by (HD), variations in values are evident in Table 1. The results highlight that the L-nov sample exhibits a higher essential oil yield (1.16%) compared to the L-mar (1.09%), St-nov (0.44%), St-mar (0.29%), and S-nov (0.69%) samples. On the other hand, the S-mar sample demonstrates the highest mass yield of essential oil (1.96%) among all the samples listed in Table 1. This comparative analysis underscores notable differences in values across the various samples, providing insights into the influence of seasonal variations on the essential oil yield of *P. nigrum* cv Guajarina. Concerning the samples extracted by steam distillation (SD), the variations in L-nov and L-mar exhibit less discrepancy in essential oil yields, at 1.37% and 1.29%, respectively. It is also possible to observe that, in this case, in addition to seasonality, the extraction method (HD) or (SD) can influence the essential oil yield of *P. nigrum* cv Guajarina. In the simultaneous distillation and extraction process (SDE), distinct yields are not obtained. This extraction method is specifically adapted to capture aromas solubilized in an organic solvent such as n-pentane. In this context, only the chemical profile of the compounds extracted by this technique can be characterized.

### 2.2. Chemical Composition

The concentration values of chemical compounds derived from *Piper nigrum* cv Guajarina plant, analyzed across different plant parts and three extraction methods, reveal diverse patterns Table 2. The dataset encompasses a broad spectrum of chemical compounds, spanning aldehydes, terpenes, and aromatic substances. Examples include (2e)-hexenal, α-pinene, limonene, linalool, and phenyl ethyl alcohol. Distinct chemical profiles emerge from different plant parts. Stems (St-mar, St-nov) exhibit higher concentrations of sabinene, limonene, and terpinolene, while leaves (L-mar, L-nov) display elevated levels of linalool and other compounds. Extraction methods play a pivotal role in determining compound concentrations. Hydrodistillation (HD) tends to yield higher concentrations for specific compounds like β-pinene, limonene, and β-caryophyllene. Simultaneous distillation and extraction (SDE) and steam distillation (SD) exhibit different extraction efficiencies for various compounds. Compounds show differences between seasons (s-nov, s-mar), suggesting a potential impact of seasonal changes on the chemical composition of essential oils. Some compounds like α-pinene, limonene, β-caryophyllene, and linalool consistently appear in different plant parts and extraction methods, indicating their importance in the overall chemical profile. Compounds like limonene and linalool, known for their aromatic and therapeutic properties, may be of particular interest to industries requiring specific chemical profiles.

In total, 122 different chemical constituents were identified in the EOs of *P. nigrum*, with sesquiterpenes being the predominant class of compounds, Table 2. The aromas were obtained by simultaneous distillation–extraction (SDE) and are listed in Table 2. The major constituents of the leaves collected in March were curzerene (23.7%), γ-elemene (23.2%), and δ-elemene (7.5%); in stems, β-caryophyllene (21.9%), curzerene (14.8%), and γ-elemene (14.1%) prevailed; in the spikes, the majority of compounds were β-pinene (22.7%), limonene (17.1%) and γ-elemene (7.5%). In November, on the other hand, limonene (19.3%), β-pinene (19.1%), and γ-elemene (8.8%) predominated.

The essential oils were obtained using hydrodistillation and steam distillation, and the chemical composition can be seen in Table 2. In hydrodistillation, the EO of the leaves collected in November (nov) was characterized by γ-elemene (32.6%), curzerene (31.2%), and δ-elemene (7.1%); in the stems there was a predominance of γ-elemene (26.8%), curzerene (20.4%) and β-caryophyllene (16.2%). The compounds γ-elemene (18%), curzerene (17.1%) and limonene (11.2%) were the main ones found in the spikes. In the EO of the leaves obtained in the month of March, the majority of the γ-elemene (34.4%), curzerene (27.4%), and δ-elemene (5.3%), we had stems γ-elemene (29.3%), curzerene (22.9%), and β-caryophyllene (13.3%); on the other hand, in the spikes there was the presence of limonene (15.15), γ-elemene (12.5%), and curzerene (20.4%).

In steam distillation, the EO of the leaves of the month of November was characterized by γ-elemene (33.9%), curzerene (31.7%), and δ-elemene (7%). By March, the majority of the following were already present: γ-elemene (31.8%), curzerene (27.1%), and δ-elemene (10.1%). The chemical profile of the essential oils differed in this study, and this variation was related to the different extraction techniques, as well as the collection periods and aerial parts of the species [7].

Preliminary studies of specimens of *P. nigrum* showed that the fresh fruits are characterized by caryophyllene (62.23%), 3-carene (26.84%), D-limonene (25.83%), caryophyllene oxide (8.17%), (−)-spathulenol (5.32%), α-copaene (5.04%), and humulene (4.13%) [14]. In another study, limonene (25.34%), sabinene (22.86%), and *β*-pinene (10.43%) characterized the EO of the fruits of this species [15]. The major compounds identified in the fine powder of the EO of fresh fruits of *P. nigrum* were *β*-caryophyllene (29.49%), 3-carene (19.20%), and limonene (18.68%) [16]. In the EOs of the leaves of *P. nigrum*, α-muurolol (20.63%), bicyclogermacrene (7.55%), cubebol (6.49%) and δ-cadinene (6.04%) were identified as the most abundant constituents [17].

*β*-Caryophyllene (21.17%), followed by δ-carene (20.23%), limonene (17.64%), *β*-pinene (14.02%), α-pinene (7.16%), myrcene (4.34%), δ-elemene (3.15%), and β-farnesene (2.16%), were the major constituents of the EO of *P. nigrum* collected in Egypt [18]. In another study, the chemical profile of the EO of *P. nigrum* obtained by supercritical carbon dioxide (SC-CO_2_) included *β*-caryophyllene (25.38 ± 0.62%), limonene (15.64 ± 0.15%), sabinene (13.63 ± 0.21%), 3-carene (9.34 ± 0.04%), β-pinene (7.27 ± 0.05%), and α-pinene (4.25 ± 0.06%). β-Caryophyllene (18.64 ± 0.84%), limonene (14.95 ±0.13%), sabinene (13.19 ± 0.17%), 3-carene (8.56 ± 0.11%), β-pinene (9.71 ± 0.12%), and α-pinene (7.96 ± 0.14%) were found in the EO obtained by HD [19]. Caryophyllene (23.98%) and limonene (14.36%) were the major constituents in the study by [6]. Another evaluation of the EO of this species by [20] demonstrated the presence of β-caryophyllene (51.12%) and β-thujene (20.58%). In another study, the EOs of fresh and ripe fruits of *P. nigrum* were characterized by the major compounds β-caryophyllene (16.0%), sabinene (12.6%), limonene (11.9%), and torreyol (9.3%) [21]. In summary, these data provide valuable insights into the chemical composition of essential oils from *P. nigrum* cv Guajarina, considering different plant parts and extraction methods. These findings contribute to our understanding of factors influencing variability in essential oil composition, with potential applications in various industries.

**Table 2:** Chemical composition of aroma, and essential oil and of *Piper nigrum* cv Guajarina. SDE: simultaneous distillation and extraction; HD: hydrodistillation; SD: steam distillation; L: leaves; St: stem; s: spike; nov: November; mar: March. Concentration results are expressed in (%).

			*Piper nigrum* cv Guajarina											
			Aroma (SDE)				Essential Oil (HD)						Essential Oil (SD)	
Constituents	* RI_L_	** RI_C_	L-mar	St-mar	s-nov	s-mar	L-nov	L-nar	St-nov	St-mar	s-nov	s-mar	L-nov	L-mar
(2E)-Hexenal	846	846	0.2											
α-Thujene	924	919		0.5		0.7						0.7		
α-Pinene	932	932	0.1	1.6	5.6	5.5		0.1			1.4	2.4		
Sabinene	969	969		5.8					0.1			6.4		
β-Pinene	974	974	0.1	0.6	19.1	22.7					8.2	9.4		
Myrcene	988	988	0.1			2						1.1		
α-Phellandrene	1002	997		0.2		0.2						0.2		
α-Terpinene	1014	1013	0.2	1	1	0.5			0.1		0.5	0.7		
p-Cymene	1020	1020	0.2						0.1					
Sylvestrene	1025	1023	0.1											
Limonene	1024	1024	0.2	2.9	19.3	17.3	0.1		0.3	0.1	11.2	15.1		
(Z)-β-Ocimene	1032	1032	0.4	1.1		1.6						1.3		
(E)-β-Ocimene	1044	1044			1.8		0.1		0.1		0.9			
γ-Terpinene	1054	1055	0.4	1.8	1.2	0.8			0.2	0.04	0.8	1.2		
cis-Sabinene hydrate	1065	1066		0.2	0.4	1.6					0.3	1.2		
Terpinolene	1086	1083	0.1	0.3	0.5	0.3			0.1		0.3	0.4		
Linalool	1095	1095	0.7	2.2	5.3	4.1	0.2	0.1	0.2		1.6	2.8		
Phenyl ethyl alcohol	1106	1104				0.03								
(Z)-p-Menth-2-en-1-ol	1118	1121		0.1	0.1	0.4					0.2	0.4		
(E)-p-Menth-2-en-1-ol	1136	1138			0.1	0.2					0.1	0.2		
Terpinen-4-ol	1174	1177	0.2	0.7	2.1	3.9			0.2	0.1	3.1	5		
α-Terpineol	1186	1191		0.1	0.8	0.7					0.5	0.5		
Methyl chavicol	1195	1195												
cis-Piperitol	1195	1200				0.1						0.1		
Nerol	1227	1220			0.1	0.2					0.1	0.1		
Methyl citronellate	1257	1257										0.1		
p-Menth-1-en-7-ol	1273	1267	0.1	0.1		0.1						0.1		
(Z)-Carvone oxide	1273	1272			0.1						0.1			
Safrole	1285	1285	0.1											
2-Undecanone	1293	1289	0.2	0.4	0.3	0.2	0.1	0.1	0.1	0.1	0.2	0.2		0.1
δ-Elemene	1335	1335	7.5		1.4	1.6	7.1	5.3	2.9	3.3	2.7	2.1	7	10.1
α-Cubebene	1345	1345	0.1	0.04		0.1	0.2	0.1	0.1	0.1	0.1	0.1	0.1	0.2
α-Ylangene	1373	1363					0.1		0.1					
α-Copaene	1374	1374	0.2	0.1	0.1	0.1	0.2	0.3	0.2	0.2	0.1	0.1	0.4	0.8
β-Bourbonene	1387	1387						0.2						
β-Elemene	1389	1389	3.2	2.1	1.1	1.1	2.9	3.5	2.7	2.5	1.9	1.5	2.8	3.5
α-Gurjunene	1409	1401	0.2	0.1	0.1	0.1	0.2	0.2		0.1	0.1	0.04	0.2	0.3
α-Cedrene	1410	1408			0.3	0.1	0.3	0.1			0.2		0.3	
β-Caryophyllene	1417	1413	1.2	21.9	4.9	6.8	0.5	1.5	16.2	13.3	10.8	7.8	0.9	0.5
(E)-α-Bergamotene	1432	1424							1.5					
γ-Elemene	1434	1425	23.2	14.1	8.8	7.5	32.6	34.4	26.8	29.3	18	12.5	33.9	31.8
β-Copaene	1430	1430												
α-Guayene	1437	1431	0.1	0.2		0.1	0.1		0.5	0.2	0.1	0.04	0.1	
6,9-Guaiadiene	1442	1437	0.1					0.2	0.2				0.2	
cis-Muurola-3,5-diene	1448	1440	0.1					0.1		0.2				
Aromadendrene	1439	1440			0.1		0.1		0.2		0.1		0.1	0.3
α-Humulene	1452	1449	0.5	1.7	0.6	0.7	0.7	0.6	2.1	1.6	1.1	0.8	0.5	0.6
cis-Cadina-1,(6),4-diene	1475	1456					0.1						0.1	
trans-Cadina-1,(6),4-diene	1475	1467											0.1	
γ-Gurjunene	1475	1475							0.3		0.1			
γ-Muurolene	1478	1478		0.1		0.3		0.2	0.2		0.1	0.1	0.2	
Germacrene D	1484	1484	3	1.3	0.6	0.7	2.4	2.6	1.5	2	1	0.9	2.2	4.1
cis-β-Guayene	1492	1485											0.3	
Curzerene	1499	1488	23.7	14.8	8.3	6.9	31.2	27.4	20.4	22.9	17.1	10.7	31.7	27.1
β-Selinene	1489	1489	1.4	2	1.1	1.2	1.7	2.2	4	3.3	2.2	1.7	1.5	1.9
(E)-Methyl-isoeugenol	1491	1491												
δ-Selinene	1492	1492												
α-Muurolene	1500	1493		0.3	0.1				0.5	0.4	0.1	0.1	0.2	0.3
β-Dihydro agarofuran	1503	1494											0.2	
(E)-Cycloisolongifol-5-ol	1513	1495	0.1											
(EE)-α-Farnesene	1505	1496		0.2	0.3	0.2	0.7	0.4	0.3	0.4	0.3	0.4	0.4	0.4
β-Bisabolene	1505	1503							0.2	0.3				
α-Cadinene	1537	1503		0.5										
trans-Calamene	1521	1506						0.1						
γ-Cadinene	1513	1509			0.1				0.2		0.1		0.2	
δ-Cadinene	1522	1513	0.7		0.5	0.2	0.8	0.7	0.9	0.7	0.5	0.3	0.8	0.8
trans-Cadina-1.4-diene	1533	1526					0.2				0.1			
Guaia-3,9-diene	1442	1530			0.1		0.3		0.6		0.1		0.4	
Selina-3.7,(11)-diene	1545	1535			0.2		0.4	0.3	0.7		0.2		0.5	
γ-Vatirenene	1546	1533	0.7	0.2		0.2		0.3		0.3		0.3		0.5
α-Vatirenene	1547	1537			0.4		0.5		0.3		0.4		0.6	
Elemol	1548	1542	2.9	0.9	1.5	0.9	1.3	1.5	0.6	1	1.2	1	1	1.4
Germacrene B	1559	1559	2.6	1.3	0.7	0.5	2.8	2.7	3.8	2.3	1.1	0.8	3.2	2.4
(E)-Nerolidol	1561	1561	1.4	0.7	0.2	0.2	0.5	0.8	0.4	0.8	0.3	0.2	0.4	0.6
Viridiflorol	1592	1573	0.3	0.1		0.1						0.1		0.1
Caryophyllene oxide	1582	1574	0.3	2	0.6	1.1	0.1	0.1	0.7	1.4	1.2	1.2		0.1
Globulol	1590	1577		0.1	0.2	0.1	0.2	0.2			0.2	0.1	0.2	0.2
β-Atlantol	1608	1602	4.9	0.2	2.1	0.1	1.4	0.2	0.8	0.2	1.8		1.2	
Dill apiole	1620	1607	0.5											
Junenol	1618	1618												
(Z)-Asarone	1616	1619												
Muurola-4,10,(14)-di-en-1β-ol	1630	1620		0.2	0.5	0.04	1.7	0.2	1	0.1	0.5		0.2	
epi-α-Cadinol	1638	1635							0.2				0.1	
α-Muurulol	1644	1639		0.3	0.1		0.3		0.4		0.2		0.1	0.6
Exalatacin	1655	1640												
β-Eudesmol	1649	1647	0.3		0.2	0.2	0.5	0.6	0.5	0.7	0.4		0.5	0.1
Attractilone	1657	1650		0.3	0.2		0.3		0.5	0.2	0.2		0.2	
Selin-11-en-4α-ol	1658	1658												
Intermedeol	1665	1659												
(E)-Asarone	1675	1675												
Eudesm-7,(11)-en-4-ol	1700	1688	0.3	0.2	0.1	0.1	0.1	0.3	0.3	0.5	0.13		0.1	0.2
2-α-Hydroxy-amorphous-4,7,(11)-diene	1760	1760			0.1				0.3		0.12			
(E)-Isovalenennol	1793	1789							0.1				0.2	
Monoterpene hydrocarbons			2.1	16	48.9	53.2	0.2	0.1	1	0.14	23.6	40.1	0	0
Oxygenated monoterpenes			1.1	3.2	8.6	9.7	0.2	0.1	0.4	0.1	5.7	9.2	0	0
Hydrocarbon sesquiterpenes			68.5	60.94	29.8	28.4	86.1	83.3	87.4	83.4	58.6	40.28	88.9	85.6
Oxygenated sequiterpenes			11.2	5.5	5.9	3.14	6.5	4.3	5.8	5.4	6.35	3	4.5	3.6
Phenylpropanoids			0.5											
Others			0.2	0.4	0.3	0.23	0.1	0.1	0.1	0.1	0.2	0.3		0.1
Total			83.6	86.04	93.5	94.67	93.1	87.9	94.7	89.14	94.45	92.88	93.4	89.3

* RI_L_, retention index in the literature [22,23]; ** RI_C_, retention index calculated from a homologous series of n-alkanes (C_8_–C_40_) in a DB5-MS column. Relative area (%) calculated based on the peak areas.

#### 2.2.1. Chemometric Analysis

**Figure 1:**
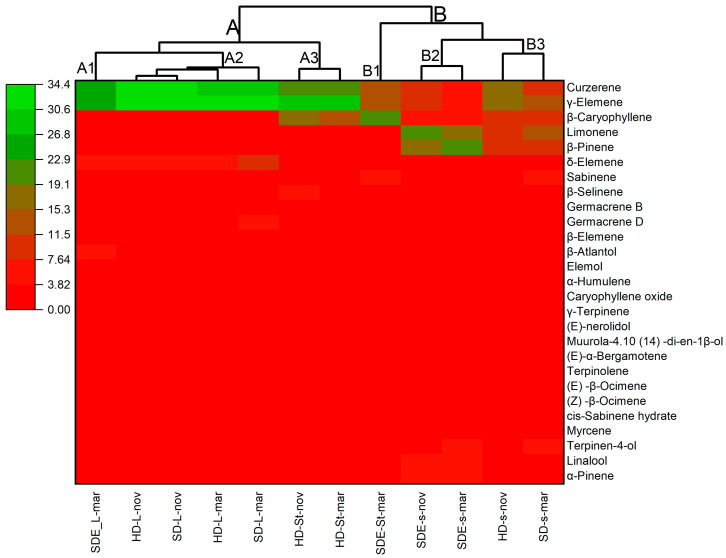
Heatmap analysis of common aroma, and essential oil constituents. The distribution of traits is identified by colors, where green indicates the maximum value of the trait, while red indicates the minimum value.

The main chemical components in the tested EOs with a relative content of more than 1% were chosen for hierarchical cluster analysis. The heatmap clustering diagram is shown in Figure 1. The heatmap is clearly divided into two main clusters, which are further divided into subclusters on the basis of their common chemical constituents. In the first cluster, there are 3 subclusters: the first subcluster represents only SDE-L-mar, whereas in the second subcluster, there are 4 samples, i.e., HD-L-nov, SD-L-nov, HD-L-mar, and SD-L-mar; the third subcluster consists of HD-St-nov and HD-St-mar. The second cluster is also divided into three subclusters, in which the first subcluster consists of SDE-St-mar only; the second subcluster has 2 samples, SDE-s-nov and SDE-s-mar; and the third subcluster also consists of 2 samples, HD-s-nov and SD-s-mar. The existence of distinct clusters indicates variations in chemical composition among the essential oil samples. The separation into subclusters further highlights specific similarities within groups. Subclusters with samples from the same source (e.g., nov or mar) suggest that there may be common chemical constituents associated with the extraction process of the essential oils.

#### 2.2.2. Principal Component Analysis

**Figure 2:**
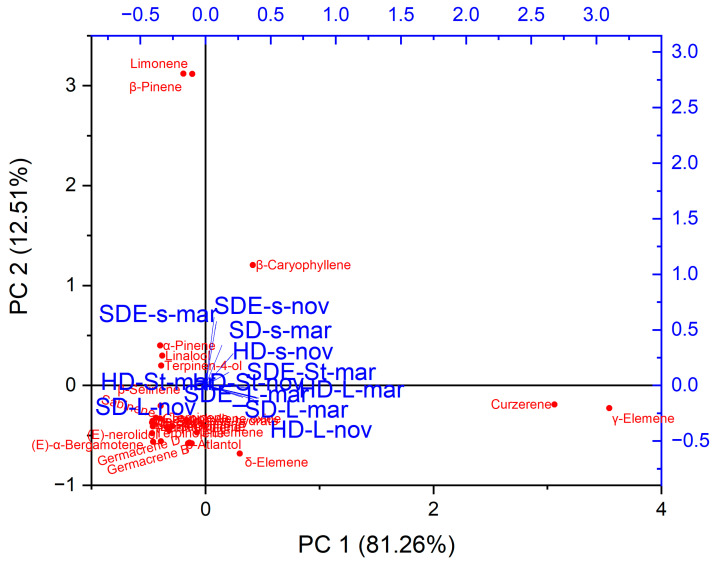
Biplot of Principal Component Analysis (PCA) for aroma (SDE) and essential oils (HD and SD) of *P. nigrum* cv Guajarina: variations in different parts of the plant and collection Times.

To assess the chemical profile changes in the EOs caused by variations in specimen and season, PCA pattern recognition was applied, as it is an important tool in multivariate statistical analysis used to identify a dataset’s most important features. The PCA determined that the first two principal components (PC1 and PC2) accounted for 93.77% of the total variance in chemical composition. PC1 was positively correlated with β-caryophyllene, which contributed 81.26% of the total variance. The strong positive correlation indicates that variations in β-caryophyllene content play a major role in explaining the differences observed in the chemical profiles of essential oils. However, PC2 contributed 12.5% of the variation and had a strong positive correlation with δ-elemene, curzerene and γ-elemene (Figure 2). Based on the PCA results, it was observed that all the essential oil samples under investigation have been grouped into four groups based on their chemical composition. In the first group, there was SDE-s-nov, SDE-s-mar, SD-s-mar, HD-s-mar, SDE-st-mar, and St-nov, and in the second group, there was HD-L-mar, SDE-L-mar, SDL-mar, and HD-L-nov. In the third and fourth groups, there was only HD-St-mar, and SD-L-nov, respectively. The HCA analysis supports the PCA results which grouped the essential oil samples into four clusters based on the relative chemical composition as similar to PCA (Figure 2).

### 2.3. Antioxidant Activity

The EOs of *P. nigrum* cv Guajarina were evaluated at a single concentration; the end point of the reaction was determined after 120 min, the absorbance was measured at 517 nm, and the results are expressed in terms of Trolox equivalents.

The tested oils exhibited DPPH inhibition ranging from 34.2 to 61.8%. As shown in Table 3, the EO of the leaves of *P. nigrum* obtained by SD in November (SD-L-nov) showed the highest antioxidant activity (167.9 mg TE mL^−1^), and its major constituents were the sesquiterpene hydrocarbons δ-elemene (7.0%) and γ-elemene (33.9%) and the oxygenated sesquiterpene curzerene (31.7%). The EO obtained by HD in March (HD-s-mar) had the next highest antioxidant activity (139.6 mg TE mL^−1^) and was characterized by the monoterpenes β-pinene (9.4%) and limonene (15.1%) and the sesquiterpenes β-caryophyllene (7.8%), δ-elemene (12.5%) and curzerene (10.7%). The high percentage of inhibition exhibited by the EO of the leaves of *P. nigrum* collected in November (SD-L-nov) may be related to the presence of sesquiterpenes because EOs rich in sesquiterpene hydrocarbon compounds generally have good antioxidant properties [24]. However, this antioxidant activity may be associated with synergistic interactions between the minor and major compounds in the EO.

Studies of the EO of *P. nigrum* corroborate that the species has promising antioxidant properties, as shown in the EO of two *P. nigrum* samples in Egypt, which were characterized by the major components β-caryophyllene (21.17–15.96%), δ-carene (20.23–27.85%), and limonene (17.64–24.07%), and showed strong oxidative inhibition in the DPPH method [18]. In another study, the EO of *P. nigrum* was characterized by the compounds β-caryophyllene (25.38–18.64%), limonene (15.64–14.95%) and sabinene (13.63–13.19%) and showed different antioxidant activity according to the DPPH method, with a higher percentage of inhibition in the EO extracted by SC-CO_2_ compared to HD [19]. This difference is directly related to the extraction method, which can affect the chemical composition of the EO, as well as the type of ecosystem, which also influences the antioxidant activity of EOs of *P. nigrum* [14].

Other studies have also shown that the EOs of *P. nigrum* have considerable antioxidant capacity [20,21,25], including anti-inflammatory potential. This correlation is due to the inflammatory processes that often function as a direct defensive response to various agents, both physical and chemical, such as oxidative stress, which is responsible for imbalances in the production of ROS and hinders the repair of damage caused by free radicals [6].

### 2.4. Preliminary Toxicity

The mortality ranged from 100% to 0% according to the concentration from 50 to 5 μg mL^−1^, as shown in Table 4.

In Table 5, it can be observed that all the oils analyzed presented a mortality rate ranging from 0 to 100%, according to the concentration of 25 to 1 μg mL^−1^. Moreover, the EO samples with the highest preliminary toxicity were obtained from the leaves (LC_50_ 6.40 µg mL^−1^) and spikes (LC_50_ 6.44 µg mL^−1^) of the species *P. nigrum* cv Guajarina in November by SD and HD, respectively. These results show that the EOs of *P. nigrum* may be promising as a natural larvicide, which is likely due to their complex composition.

The oil obtained from the leaves (SD-L-nov) predominantly contained the sesquiterpene hydrocarbons δ-elemene (7.0%) and γ-elemene (33.9%) and the oxygenated sesquiterpene curzerene (31.7%). The major compounds in the oil of the spikes (HD-s-nov) were the monoterpenes β-pinene (8.2%) and limonene (11.2%) and the sesquiterpenes β-caryophyllene (10.8%), γ-elemene (18.0%), and curzerene (17.1%). It is important to mention that natural products are toxic to Artemia species [26], and thus natural compounds have great potential in the advancement and development of low-cost ecological biopesticides [27]. Incidentally, the sesquiterpene compounds γ-elemene, curzerene, and β-caryophyllene are described in the literature as presenting potential toxicity, as are the monoterpene compounds β-pinene and limonene [28,29,30,31].

EOs from within the genus *Piper* have exhibited effective toxicological properties, as described for the EOs of several species of *Piper* collected in different regions of the Amazon: *P. aduncum*, *P. marginatum*, *P. divaricatum*, and *P. callosum* [32], as well as the EOs of those collected in Indonesia [33], and *P. alatipetiolatum* from the state of Amazonas, Brazil [34], both of which were effective in toxicity tests. These results, specifically in the Amazon region, reinforce the great importance of studies aimed at controlling several vectors since the region is a nursery of bioactive compounds with biological activities. There is a potential correlation between antioxidant potential and preliminary toxicity, as evidenced in Table 3. Remarkably, the (SD-L-nov) essential oil sample demonstrated the highest antioxidant potential, resulting in a proportionally higher value of preliminary toxicity. This direct association can be attributed to the major compounds identified in this fraction of essential oil, such as γ-elemene and curzerene. Literature consulted also points out that these compounds have antioxidant potential and have toxicity [35,36,37].

### 2.5. In Silico Study

#### Evaluation of the Interactions of Major Compounds with AChE

**Figure 3:**
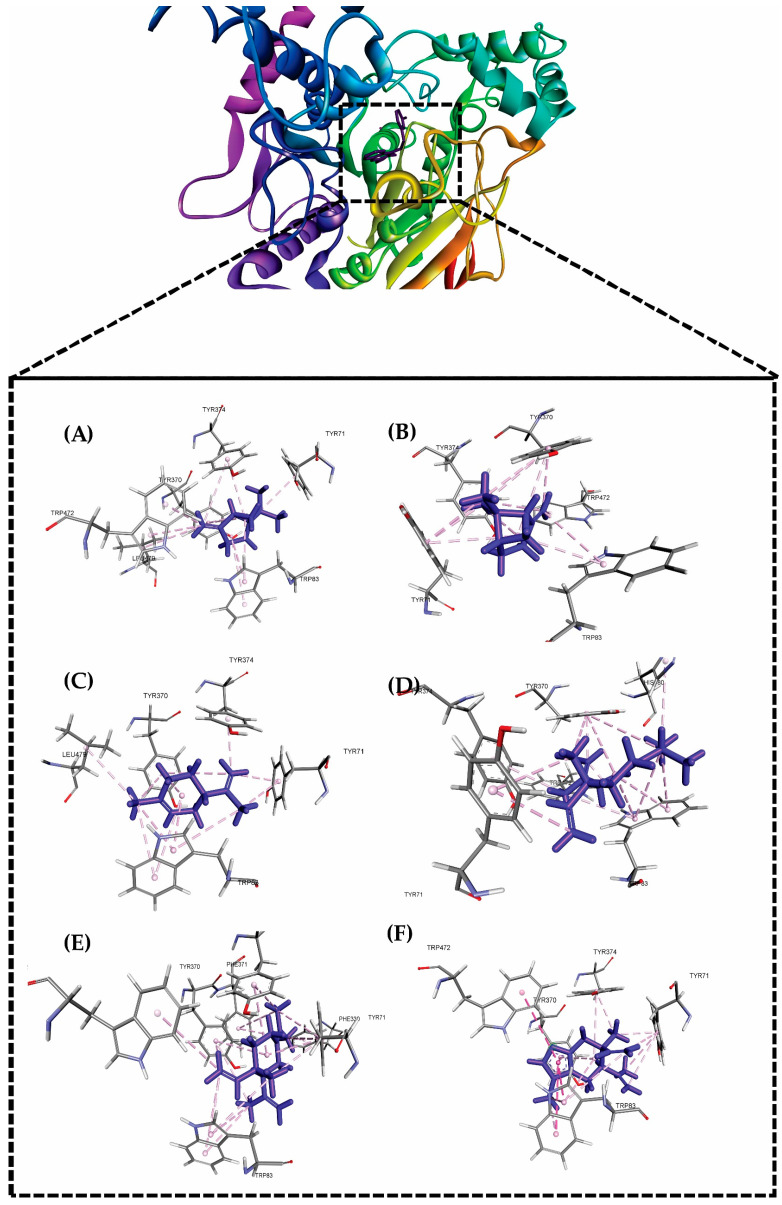
Molecular docking (3D-interaction diagram) analysis for complexes of ligands (**A**) Sabinene, (**B**) β-Pinene, (**C**) Limonene, (**D**) δ-Elemene, (**E**) γ-Elemene and (**F**) Curzerene with AChE.

In the past few decades, in silico methods have gained much popularity due to their acceptable success rates, while identifying hit compounds against particular disease targets [38]. In present study, we analyzed the possible interactions of major EO (essential oil) compounds of the selected plant with the target AChE (Acetylcholinesterase). With reference to earlier reported studies, we noticed that EO components had strong binding affinities for AChE enzyme and also, it was investigated in *A. salina* models. Before proceeding for the molecular docking simulations, and molecular dynamics analyses, we redocked the co-crystalized ligand, 9-(3-iodobenzylamino)-1,2,3,4-tetrahydroacridine into the same binding pocket of the target AChE [38]. The RMSD value was found to be <2 Å, which also indicated that the docking protocol used herein, was proper, and validated. Molecular docking simulations were used in order to assess the probable binding modes of major EO components against the AChE. Figure 3 elaborates on 3D-interaction diagrams obtained for major EO components along with their interacting amino acid residues. We had also noticed that all EO components remained interacting throughout the entire simulation periods. Sabinene interacted mainly with amino acid residues such as Tyr A:71, Glu A:80, Tyr A:374, Met A:476, Trp A:472, Tyr A:370, and His A:480, etc. (docking score: −8.32 kcal/mol). Main interaction types were of π-alkyl, alkyl and van der Waals forces. β-Pinene (docking score: −7.98 kcal/mol) remained bound within active site of the protein by forming key interactions with Tyr A:374, Tyr A:370, Trp A:472, and Trp A:83. Limonene (docking score: −9.02 kcal/mol), δ-Elemene (docking score: −9.04 kcal/mol), γ-Elemene (docking score: −9.76 kcal/mol) and Curzerene (docking score: −5.34 kcal/mol) interacted with amino acids such as Tyr A:71, Glu A:80, Tyr A:374, Met A:476, Trp A:472, Tyr A:370, and His A:480 through alkyl-type interactions or hydrophobic interactions. The best docking score was noted for the γ-Elemene (docking score: −9.76 kcal/mol).

**Figure 4:**
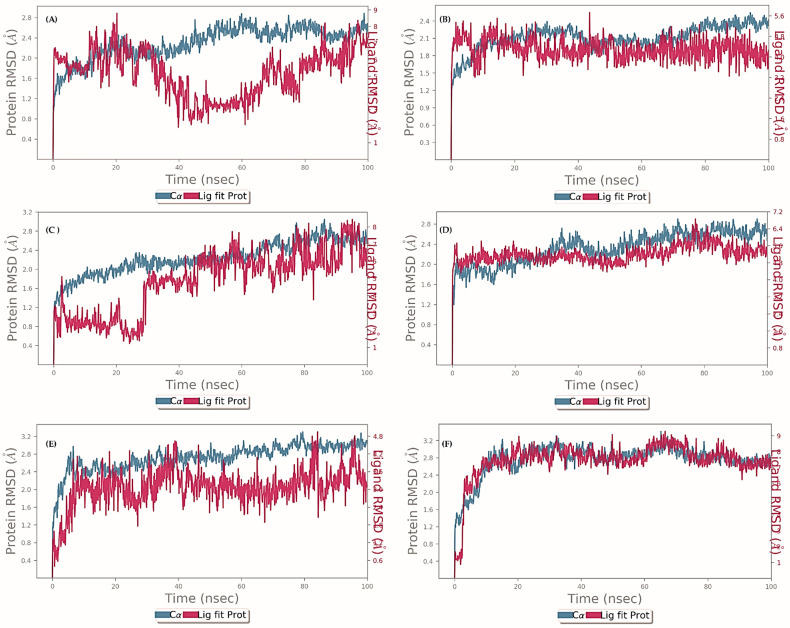
Root-mean-square deviation (RMSD) analysis for complexes of ligands (**A**) Sabinene, (**B**) β-Pinene, (**C**) Limonene, (**D**) δ-Elemene, (**E**) γ-Elemene and (**F**) Curzerene with AChE over simulation period of 100 ns.

**Figure 5:**
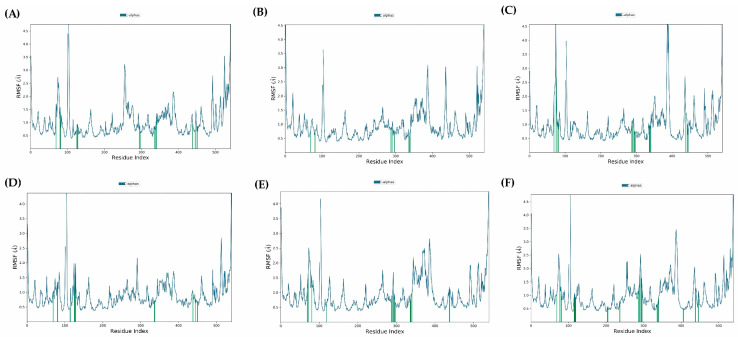
Root-mean-square fluctuations (RMSF) analysis for complexes of ligands (**A**) Sabinene, (**B**) β-Pinene, (**C**) Limonene, (**D**) δ-Elemene, (**E**) γ-Elemene and (**F**) Curzerene with AChE over simulation period of 100 ns. The green line indicates the localized changes in proteins while blue indicates actual fluctuations.

The stability and functionality of a ligand–protein complex depend on the folding and movement of the structure’s backbone. One way to gain insight into these structural deviations is through the use of root-mean-square deviation (RMSD) analysis during a 100 ns MD simulation (Figure 4). Over the period of 100 ns simulation, we analyzed 5 protein–ligand complexes with EO components as Sabinene-AChE, β-Pinene-AchE, Limonene-AchE, δ-Elemene-AchE, γ-Elemene-AchE and Curzerene-AChE. For the Sabinene-AChE complex, many fluctuations were observed over the entire simulation period retaining final RMSD below 2.8 Å. For other complexes, stabilities were achieved roughly after 40 ns of simulation time. Nevertheless, RMSD values for all simulated complexes were retained below 3.2 Å, indicating good ligand-protein stability. On the other hand, the root-mean-square fluctuation (RMSF) analysis examines the fluctuation of protein residues within the ligand–protein complex over a 100 ns period. In this study, the RMSF plot of Sabinene-AChE, β-Pinene-AChE, Limonene-AChE, δ-Elemene-AChE, γ-Elemene-AChE and Curzerene-AChE was analyzed for regions displaying higher flexibility. Figure 5 illustrates that the binding of compounds did not significantly alter the overall conformational diversity of the protein complexes. Therefore, the RMSF analysis indicates that the binding of ligands to proteins does not cause substantial changes in the reference protein residues, resulting in stable protein–ligand complexes. According to the MM/GBSA results, the ligands (A) Sabinene, (B) β-Pinene, (C) Limonene, (D) δ-Elemene, (E) γ-Elemene, and (F) Curzerene exhibited affinity towards AChE, as evidenced by their respective affinity energy values of −17.93, −29.55, −26.29, −35.69, −42.33, and −30.80 kcal/mol. These results suggest that the complexes between the ligands and AChE were spontaneously formed.

## 3. Materials and Methods

### 3.1. Collection of Botanical Material

The specimens (*Piper nigrum* cv Guajarina) were collected in the morning at the Empresa Brasileira de Pesquisa Agropecuária (EMBRAPA) located in Belém, Pará. The collection dates ranged from November 2018 to March 2019. Specimen preparation, registration, incorporation into the herbarium and identification of the studied species were performed according to traditional botanical techniques. Botanical identification was performed by comparison with materials identified by Dr. Elsie Franklin Guimarães, a specialist in Piperaceae, and samples were incorporated into the IAN Herbarium, from the botanical laboratory of Embrapa Amazônia Oriental, Belém, Pará, with voucher number Nid 111/2023.

### 3.2. Determination of Residual Moisture

Prior to moisture analysis, the sample underwent drying in an air circulation oven maintained at approximately 35 °C for a duration of 5 days. Subsequently, the moisture content of the samples was quantified utilizing a Gehaka infrared moisture analyzer IV2500 (Gehaka, São Paulo, Brazil).

### 3.3. Essential Oil Extraction

#### 3.3.1. Hydrodistillation

For the EO extraction process, 40 g of fresh botanical material was dried in an air circulation oven and then subjected to hydrodistillation (HD). The proportion of water relative to the amount of plant material was kept constant. The extraction process lasted for 10,800 s at a temperature of 100 °C. Following extraction, anhydrous sodium sulfate (Na_2_SO_4_) was introduced, and the essential oil (EO) underwent centrifugation to remove any residual moisture. The mass yield of the essential oil was computed on a dry basis (db) by correlating the oil mass obtained through hydrodistillation (HD) with the dry mass employed in the extraction process.

#### 3.3.2. Simultaneous Distillation and Extraction

To extract the aroma, simultaneous distillation–extraction methods were employed using a Chrompack Nickerson & Likens extractor, which was connected to a refrigeration system (5–10 °C) and linked to two round-bottomed flasks. In this process, 10 g of botanical material and 125 mL of distilled water were introduced into a 250-mL flask equipped with a heating mantle. The vapors produced in this flask passed through the condenser. In a separate 5 mL flask, 2 mL of n-pentane were added and placed in a water bath maintained at 53–56 °C, facilitating the evaporation and extraction (condensation) of the aroma. The extraction duration was set at 2 h.

#### 3.3.3. Steam Distillation

Steam distillation (SD) was conducted using an adapted Clevenger glass apparatus connected to a refrigeration system, ensuring that the condensation water remained within the temperature range of 10 to 15 °C over a duration of 3 h. Following the extraction process, the obtained oils underwent centrifugation for 5 min at 3000 rpm and subsequent dehydration using anhydrous Na_2_SO_4_. The oil yield was determined as a percentage (%). To preserve the oils, they were stored in flame-sealed amber glass ampoules in a refrigerator at 5 °C.

### 3.4. Identification of Chemical Constituents

The chemical compositions of the essential oils (EOs) were assessed utilizing a single quadrupole gas chromatography/mass spectrometry (GC/MS) system (Thermo DSQ-II, Waltham, MA, USA) equipped with a DB-5MS silica capillary column (30 m × 0.25 mm, 0.25 mm; Agilent Technologies, Stevens Creek Blvd. Santa Clara, CA, USA). The analytical conditions involved a temperature gradient from 60 to 240 °C at a rate of 3 °C/min, with the injector temperature set at 240 °C. Helium served as the carrier gas (linear velocity of 32 cm/s, measured at 100 °C), and a one-step injection of aqueous 2:1000 n-hexane (0.1 µL) was employed. The temperature of the ion source and other components was maintained at 200 °C. The quadrupole filter was scanned in the range of 39–500 Da every second, utilizing an electron impact technique at 70 eV for ionization. Retaining consistency with the Van den Dool and Kratz [39] method, the retention index for all volatile constituents was computed using a homologous series of n-alkanes (C_8_–C_40_) (Sigma–Aldrich, San Luis, AZ, USA). Identification of components was accomplished by comparing (i) the experimental mass spectra with those compiled in libraries (reference) and (ii) the retention indices with those documented in the literature [22,23]. Quantification of the volatile constituents was performed through peak-area normalization, utilizing a FOCUS GC/flame ionization detector (FID) operating under the same conditions as the GC–MS instrument, with the exception of the carrier gas, which was nitrogen.

### 3.5. Determination of Preliminary Toxicity in Artemia salina Leach

An artificial brine solution was meticulously prepared by dissolving 46 g of NaCl, 22 g of MgCl_2_·6H_2_O, 8 g of Na_2_SO_4_, 2.6 g of CaCl_2_·2H_2_O or CaCl_2_·6H_2_O, and 1.4 g of KCl in 2000 mL of distilled water. To ensure a stable environment for larval incubation, the pH of the brine was precisely adjusted to 9.0 using Na_2_CO_3_, mitigating the risk of larval mortality attributed to pH fluctuations during the incubation period. *A. salina* cysts (25 mg) were carefully incubated in the artificial brine solution at a temperature of 25 °C within a glass container boasting a volume capacity of 10.6 dm^3^. This container featured an oxygenation system facilitated by an aeration pump. Notably, the glass container was partitioned into two sections: one housing the eggs, shielded from light, and the other exposed to artificial illumination emitted by a 40 W lamp. This partition ensured the separation of larvae post-hatching, as they exhibit positive phototropism, gravitating towards light. The essential oil (EO) solution was meticulously prepared, attaining a concentration of 1250 μg mL^−1^, utilizing brine water (devoid of larvae) as the solvent and 5% dimethyl sulfoxide (DMSO) as the solubilizer. From the stock solution, aliquots were methodically diluted to varying concentrations: 1, 5, 10, 25, 50, 100, 250, 500, and 1000 μg mL^−1^. Twenty-four hours post-hatching, approximately 10 larvae were introduced into individual sample test tubes via an automated micropipette. These tubes were then filled to a total volume of 5 mL with brine water. The control group consisted of 5 mL of 5% DMSO brine harboring 10 *A. salina* larvae. Notably, all experiments were meticulously conducted in triplicate (n = 3). Following 24 h of interaction between the *A. salina* larvae and the sample solutions, the percentage mortality was meticulously calculated. Subsequently, the LC50 value was determined through semilogarithmic interpolation, converting mortality percentages into probits. As a positive standard for the control group, lapachol, a naphthoquinone extracted from the bark of various *Tabebuia* species (Bignoniaceae), renowned for its broad biological activity against diverse organisms, was employed [40].

### 3.6. Antioxidant Potential

The essential oil samples (10 μL) were combined with 900 μL of 100 mM Tris-HCl buffer (pH = 7.4), 40 μL of ethanol, and 50 μL of a 0.5% Tween 20 solution (m/m). Following this, 1.5 mL of 0.5 mM DPPH in ethanol (250 μM in the reaction mixture) was introduced. Tween 20 acted as an emulsifier to facilitate oil–water mixing. The mixture underwent vigorous stirring and was incubated in a dark environment at room temperature for 30 min. Subsequently, absorbance readings were taken in the UV-visible range at 517 nm using an 800XI spectrophotometer (Femto; São Paulo/SP, Brazil). For the control reaction, 50 μL of Trolox 1 mM in ethanol was substituted for the sample (resulting in a final concentration of 25 μM in the reaction). The calculation of the inhibition percentage (DPPH %) was performed as described in the literature to determine the percentage of inhibition of DPPH radicals (DPPH) [40].

### 3.7. Molecular Modeling Study

#### In Silico Analysis (Molecular Docking and Molecular Dynamics)

Exploring the interactions of six different chemical compounds ((A) sabinene, (B) β-pinene, (C) limonene, (D) δ-llemene, (E) γ-elemene and (F) curzerene) with the active site of the acetylcholinesterase (AChE) enzyme, we conducted a series of molecular docking experiments. The molecular structures were visualized using ChemDraw, and optimization was carried out through the MM2 force field. Autodock Vina was employed for the docking simulations, utilizing the X-ray crystallographic structure of BuChE retrieved from the PDB. Thermodynamic stability was assessed by calculating binding affinities, and graphical representations were generated using PyMOL and VMD. Subsequently, we delved into molecular dynamics simulations of the docked complexes using the NAMD software 2.14. The CHARMM36 force field and TIP4P water model were employed in an implicit solvent environment. Counter ions were introduced to neutralize the system, and to mimic physiological conditions, saline solutions were included. The equilibration process encompassed NVT and NPT ensembles for 15 ns each, employing a Langevin thermostat and barostat. A time step of 2 fs was maintained during the simulations, and the final production run was extended for 120 ns. Monitoring parameters included RMSD, gyration radius, RMSF, hydrogen bonds, electrostatic interactions, and solvent-accessible surface area calculations [41,42]. The compounds were drawn using GaussView 6 and optimized via B3LYP/6-31G* using Gaussian quantum chemistry software 16 [38,43] and the Glide software V. 2023 from Schrodinger, LLC, 2023 [44,45]. Next, molecular dynamics simulations were carried out on the docked complexes using the Desmond 2022 software V. 2023, from Schrodinger, LLC. The OPLS-2005 force field [46,47] and TIP3P water compounds were used in an explicit solvent model [48].

### 3.8. Statistical Analysis

Chemometric analysis, integrating heatmap analysis and principal component analysis (PCA), was performed using OriginPro, version 2023. This analysis was applied to aroma (SDE) and essential oils (HD and SD) extracted from *P. nigrum* cv Guajarina, considering variations between different parts of the plant and collection times. Heatmap clustering was used to show intricate patterns and correlations, while PCA was used for a comprehensive visualization of variation and underlying structure within the dataset.

## 4. Conclusions

This study provides significant insights into the composition of the essential oil (EO) from *P. nigrum* cv Guajarina, highlighting the impact of extraction techniques and seasonal variations on the observed differences. The highest EO yield was obtained through hydrodistillation (HD) during the rainy season, revealing distinct profiles in leaves, stems, and spikes. The prevalence of terpene substances, particularly hydrocarbon sesquiterpenes such as Sabinene, β-pinene, limonene, δ-elemene, γ-elemene, and curzerene, underscores the diversity in the chemical composition of the oils. Additionally, the antioxidant activity of the EOs, a crucial factor in their potential applications, showed notable variations depending on the collection time and plant part. The EOs extracted from leaves during the dry season and spikes in the rainy season of *P. nigrum* cv Guajarina exhibited the highest antioxidant activity, containing key compounds such as δ-elemene, γ-elemene, curzerene, β-pinene, limonene, and β-caryophyllene. It is important to recognize that the observed variations in the major compounds of EOs have multifactorial origins, possibly related to genetic factors and seasonal fluctuations. Moreover, the in silico study demonstrated that the major compounds may be associated with the potential toxic effect on *A. salina*, with the primary molecule being the enzyme acetylcholinesterase.

## Figures and Tables

**Table 1:** Yields of essential of *Piper nigrum* cv Guajarina oils extracted by different methods, HD: hydrodistillation; SD: steam distillation; L: leaves; St: stem; s: spike; nov: November; mar: March. EO: Essential oil. The yield values are expressed in (%).

*Piper nigrum* cv Guajarina									
(HD)							(SD)		
Yield (%) EO	L-nov	L-mar	St-nov	St-mar	s-nov	s-mar	L-nov	L-mar	
	1.16	1.09	0.44	0.29	0.69	1.96	1.37	1.29	

**Table 3:** Antioxidant potential of different fractions of essential oil of *P. nigrum* cv Guajarina, HD: hydrodistillation; SD: steam distillation; L: leaves; St: stem; s: spike; nov: November; mar: March. Inhibition values were measured in (%).

Samples (EOs)	Inhibition (%)	mg TE mL^−1^
SD-L-nov	61.8 ± 4.2	167.9 ± 11.5
SD-L-mar	34.2 ± 2.8	93.1 ± 7.5
HD-L-mar	40.3 ± 2.27	109.5 ± 7.3
HD-s-mar	51.4 ± 1.9	139.6 ± 5.2

**Table 4:** Concentration–response relationship for the lapachol standard and LC_50_ value, using *Artemia salina* as a model.

Sample	Concentration (µg mL^−1^)	Mortality (%)	R^2^	LC_50_
	50	100		(µg mL^−1^)
Lapachol	25	66.7		
	10	3.3	0.93	21.2 ± 2.2
	5	0		

**Table 5:** Preliminary toxicity in *Artemia salina* of different fractions of essential oil from *Piper nigrum* cv Guajarina, HD: hydrodistillation; SD: steam distillation; L: leaves; St: stem; s: spike; nov: November; mar: March.

Sample	Concentration (µg mL^−1^)	Mortality (%)	R^2^	LC_50_ (µg mL^−1^)
SD-L-nov	25	100		
	10	76.6	1	6.40 ± 0.26
	5	43.3		
	1	0		
SD-L-mar	25	100		
	10	63.3	1	7.25 ± 0.05
	5	13.3		
	1	0		
HD-L-nov	25	100		
	10	26.6	0.9	7.95 ± 0.15
	5	16.6		
	1	0		
HD-L-mar	25	100		
	10	60	1	7.22 ± 0.26
	5	16.6		
	1	0		
HD-s-nov	25	100		
	10	76.6	1	6.44 ± 0.26
	5	40		
	1	0		
HD-s-mar	25	100		
	10	80	1	6.65 ± 0.11
	5	23.3		
	1	0		

## Data Availability

Data is contained within the article.
